# Dr. Indira Nath: Innovator in Leprosy Immunology and a Beacon of Scientific Excellence

**DOI:** 10.7759/cureus.67796

**Published:** 2024-08-26

**Authors:** Barani R Selvarasu, Nishikant Ingole

**Affiliations:** 1 Department of Pharmacology, Jawaharlal Nehru Medical College, Datta Meghe Institute of Higher Education and Research, Wardha, IND

**Keywords:** historical vignette, international assignments, cancer, leprosy, autoimmune condition, immunology

## Abstract

Dr. Indira Nath, an Indian immunologist, was born on January 14, 1938 and passed away on October 24, 2021. Dr. Indira Nath is considered one of the pioneers of immunology in India. Immunologists study the role of the immune system in human health and diseases. While many immunologists prefer to study cancers and autoimmune conditions, in contrast, Dr. Indira Nath chose to study the immunology of leprosy, a neglected tropical disease that had a considerable disease burden and social stigma in India. She returned to India and joined her alma mater, where she continued the rest of the journey until her formal retirement. Meanwhile, Dr. Nath also oversaw the establishment of a new department of biotechnology at the All India Institute of Medical Sciences (AIIMS), New Delhi, where she served as the founding head and further advanced her new-age research on the immunology of leprosy.

## Introduction and background

Dr. Indira Nath completed her medical education, Bachelor of Medicine and Surgery (MBBS) and Doctor of Medicine (MD), from the prestigious AIIMS, New Delhi, and specialized in immunology from the United Kingdom as a Nuffield fellow. She returned to India and joined her alma mater where she continued the rest of the journey until her formal retirement. Meanwhile, Dr. Nath also oversaw the establishment of a new Department of Biotechnology at AIIMS, where she served as the founding head and further advanced her new-age research on the immunology of leprosy. However, even two decades after retirement, she was still active in immunological research. Indira Nath's research on leprosy has significantly contributed to understanding the disease and treatment. The main focus of Indira Nath's research has been on the Rapid, Radiolabeled-Microculture Method Using Macrophages for In Vitro Evaluation of *Mycobacterium leprae* Viability and Drug Susceptibility. This study describes a fast microculture approach that uses radiolabeling and mice macrophages to determine the medication resistance or helplessness of *Mycobacterium leprae* as well as its reasonableness. Defenselessness or protection against dapsone was revealed by analyses of *Mycobacterium leprae* occupant macrophage societies kept in 96-well-level lined plates, and the feasibility results were comparable to those of concurrent societies in Leighton tubes with greater concentrations of bacilli and macrophages. She has researched the mechanisms by which the distinctive symptoms of leprosy develop, as well as how the disease progresses from the initial infection to more severe forms. Nath has been engaged in endeavors to foster successful immunizations against infection. This includes looking into potential candidates for vaccines and learning how they can trigger a protective immune response. She has also been credited with the sequencing of the LSR2 gene in leprosy-causing bacteria. Her research also guided experiments on experimental immunotherapy treatments and candidate vaccines for leprosy. Her research fetched her national as well as international recognition as an expert on the immunology of leprosy. Therefore, she was also able to advise the governments on the strategies adopted in leprosy control programs [1,2].

## Review

Early life and career

Educational Track

Dr. Indira Nath was born on January 14, 1938, in Guntur, Andhra Pradesh. The eldest daughter of Smt. Nagalaxmi and Shri N.V. Rao, a Central Public Works Department (CPWD) engineer. She had her early schooling at La Martiniere in Kolkata. She did her pre-medical course (Hindu College) and was selected for the MBBS in 1957 at AIIMS (second batch). After completing the MBBS course in 1961 (gold medalist), she specialized in pathology (MD, 1969). During her Nuffield Postdoctoral Fellowship (1971), she honed her skills in the in vitro culture and maintenance of murine macrophages. Her long-standing connections with Teacher John Turk at the illustrious School of Specialists, as well as her improved examination experience with Dr. R.J.W. Rees of the Public Establishment for Clinical Exploration in London, UK, have been invaluable. She was appointed as a lecturer at the AIIMS Department of Biochemistry upon her return in 1972. She chose to work in the medical field because of her underlying openness to filthy patients at the Shahdara State when she was an undergraduate. She joined the faculty at AIIMS after immunology was added as a new department, and she immediately expressed interest in researching the immunological elements of human leprosy [3].

Work Experience

In the 1970s, she returned to the AIIMS in New Delhi after serving as a house officer in several UK hospitals. Once Nath had lost faith in pathology, he became enthralled with the immunology of disease during a year-long Nuffield Partnership at the Public Establishment of Clinical Exploration in London. The field of immunology began with the discovery of T- and B-cells. The World Health Organization (WHO) created multidrug treatment (MDT) for leprosy, which consists of dapsone, clofazimine, and rifampin. The WHO is now willing to tolerate a prevalence of 1:10,000 and has altered the phrase to "elimination." The population prevalence rate in India is approximately 5.7 per 10,000 [4].

Leprosy Bacillus

Leprosy has been known since ancient times and was first identified by H. Armauer Hansen in 1873. *Mycobacterium leprae* is the organism that causes it. Traditionally associated with filth, leprosy mostly affects the skin and nerves. Leprosy was found in a person buried close to Jerusalem between 1 and 50 AD, according to DNA evidence. The disease most likely originated in Egypt and the Middle East around 2400 BCE. Due to the WHO's implementation of multidrug therapy (MDT), in 90% of endemic nations, the incidence of leprosy has dramatically declined during the past 20 years to less than one case per 10,000 individuals [5].

Research

Her research focuses on the disease's nerve damage and cellular immune responses in humans with leprosy. Her work has likewise searched for marks of the disease bacillus making due. She has north of 120 distributions, welcomed surveys, and assessments/remarks on late advancements in global diaries [6,7].

Work on the Infection of Macrophages With Mycobacterium leprae to Examine Gene Activation

Leading promises of cell resistance in human filth and the release of Lsr2 quality in sick bacillus were made by Professor Nath. She demonstrated the clear availability of CD4+FOXP3+T administrative cells that produce transforming growth factor alpha (TGF-α), which could explain the specific antigen energy associated with persistent lepromatous uncleanliness. However, her focus was on investigating the phone theory that some patients (temperamental) have "reactions" that result in severe depression and necessitate immediate medical attention. In a series of publications, she and Dr. Chaman outlined a new paradigm that goes beyond the traditional Th1 and Th2 T-cell subsets: patients with leprosy responses have an imbalance in the Th17 and Treg populations. An imbalance resulted from changes in cytokine expression, including increased IL-6 and IL-21 and inhibition of TGF-α. Th17's migration to flammable locations was aided by the expanded articulation of chemokines (CCL20 and CCL22). An increase in hyperactive Th17 cells at the lesional sites may worsen the immunopathology and inflammation observed in leprosy reactions [8,9,10].

Immunology of Leprosy

Nath's research significantly advanced the understanding of the immune responses involved in leprosy. She studied the cellular and molecular mechanisms underlying the disease, focusing on how the body's immune system interacts with the *Mycobacterium leprae* bacterium, which causes leprosy. Her work helped identify the various immune responses that determine whether a person will develop a severe form of the disease or a milder form [11].

Immunopathology

Nath explored the pathogenesis of leprosy, investigating how the disease progresses and the factors that influence its severity. She gained insight into the immunopathology of leprosy by studying how various immune cells, such as T-cells and macrophages, contribute to the disease's progression [12].

Development of Diagnostic Tools

Nath's research contributed to the development of better diagnostic tools for leprosy. Her work helped in the identification of biomarkers that could be used to diagnose the disease more accurately and to monitor its progression and response to treatment. [13]

Vaccination and Treatment Strategies

She was involved in research aimed at developing vaccines and improving treatment strategies for leprosy. Her studies on the immune responses in leprosy patients provided a foundation for developing potential vaccines and therapeutic approaches [14].

Interdisciplinary Research

Nath's work often involved interdisciplinary approaches, combining immunology, microbiology, and clinical research. She collaborated with scientists and clinicians worldwide to address the challenges posed by leprosy and other infectious diseases [15].

On Leprosy

In an interview that aired on India's state TV, Doordashan's Eureka program, Indira stated that she was never impacted by the stigma associated with leprosy. She also notes that the leprosy insect is not lethal, characterizing it as a cunning bug that merely seeks a tranquil existence within the body. "So we should look at it kindly." "Leprosy is not contagious at all," she stated. Infections like the flu and the cold are far more common. The leprosy bug does not spread quickly and grows very slowly. Years pass during the incubation period." According to her, patients are scared because of the nerve damage and physical abnormalities they observe. [16]

Distinction

She was elected Fellow of the National Academy of Sciences, Allahabad (1988), Indian Academy of Sciences, Bangalore (1990), Indian National Science Academy (INSA; 1992), National Academy of Medical Sciences (India) (1992), Royal College of Pathology (1992) and the Academy of sciences for the developing world (TWAS) (1995). She was a member of the Scientific Advisory Committee to Cabinet, Foreign Secretary of INSA (1995-97), Council Member (1992-1994, 1998-2006), and Vice President (2001-2003) of the National Academy of Sciences (India), Allahabad, and chairperson, Women Scientists Programme, Department of Science and Technology, India (2003) [17].

Awards

Besides her contributions to leprosy, she was also recognized for excellence in research ethics and global health in her over five decades-long career and involved herself in various global health activities. For her research and contributions, she was awarded the prestigious Padma Shri and Shanti Swarup Bhatnagar Awards by the Government of India. She was also given the civilian title of Chevalier Ordre National du Mertie by the French government. In addition, she received recognition for her achievements with the Cochrane Research Award and the L'Oreal United Nations Educational, Scientific, and Cultural Organization (UNESCO) Award for Women in Science [18].

Journey End

On October 25, 2021, the Disease Mission Trust India Exploration Board was supposed to meet via Zoom, with executive Indira Nath present. Regretfully, there was no gathering. The previous evening, Professor Indira Nath had passed away peacefully. The hopeless misfortune, a sensation of gloom, plummeted on all who were special to know her. Professor Indira Nath's demise has left us without a distinguished biomedical scientist. Professor Nath is recognized internationally for his contributions to our knowledge of the cellular immune responses of humans with leprosy. Throughout her more than 50-year career, Professor Indira Nath was a leading authority on research ethics, international scientific collaboration, and public health globally. She infused clinical and educational strategies with fresh teachers and her signature vigor and excitement to bring about change [19].

## Conclusions

Dr. Indira Nath's exceptional excursion in immunology is a demonstration of her significant effect on the field, especially in the investigation of uncleanliness. Brought into the world in 1938 in Guntur, Andhra Pradesh, her distinguished lifetime started with clinical schooling at AIIMS Delhi and further specialization in immunology in the UK. She established and led the Department of Biotechnology at AIIMS upon her return to India, where she carried out pivotal research on the immunology of leprosy. We now know a lot more about leprosy thanks to Nath's ground-breaking research on the disease's cellular immune responses and the Rapid, Radiolabeled-Microculture Method she developed. Her examinations uncovered significant experiences in the sickness systems, including the revelation of the Lsr2 quality and the job of Th17 and Treg cells in uncleanliness responses. Her efforts in vaccine development and experimental immunotherapy have furthered the field's understanding of leprosy. Through her vocation, numerous awards, including the Padma Shri and Shanti Swarup Bhatnagar Awards, as well as international honors like the Chevalier Ordre National du Mérite from France, were bestowed upon Nath. Her work procured her a spot among the premier researchers universally, with an inheritance set apart by her obligation to explore morals, general well-being, and logical cooperation. Nath's passing in October 2021 denoted the conclusion of a significant period for disease research. Her groundbreaking discoveries and profound influence on immunology have left a lasting legacy. She abandons a tradition of logical greatness and devotion to reducing one of the world's most disregarded illnesses, encapsulating the soul of request and empathy all through her recognized profession.
